# WMC-DFINE: An Improved DFINE Model for Aluminum Profile Surface Defect Detection

**DOI:** 10.3390/s26102994

**Published:** 2026-05-09

**Authors:** Pengfei He, Yunming Ding, Shuwen Yan, Guoheng Wang, Xia Liu

**Affiliations:** 1 School of Physics and Electronic Information, Yantai University, Yantai 264005, China; hpf_972@ytu.edu.cn (P.H.); dyunming0902@s.ytu.edu.cn (Y.D.); ytuyanshuwen@s.ytu.edu.cn (S.Y.); 2 Shandong Provincial Laboratory of Data Open Innovation and Application for Advanced Smart Grid Technologies, Yantai University, Yantai 264005, China; 3 Yantai Dongfang Wisdom Electric Co., Ltd., Yantai 264005, China; wangguoheng@dongfang-china.com

**Keywords:** aluminum profile surface defect detection, DFINE, wavelet transform, cross-scale feature fusion, knowledge distillation

## Abstract

The automated inspection of aluminum profile surface defects, which heavily relies on data acquired by machine vision sensors, is a critical task in industrial quality control. Addressing the current challenges of intense background texture interference and the difficulty in detecting defects with extreme aspect ratios on aluminum profiles, this research puts forward a complete end-to-end defect detection algorithm named WMC-DFINE (WIFA-MKSS-CSFF-DFINE) based on the DFINE framework. First, a Wavelet-Integrated Frequency Attention (WIFA) module is introduced, which utilizes a discrete wavelet transform to decouple features into the frequency domain, thereby dynamically suppressing high-frequency background noise and enhancing defect edge responses. Second, a Cross-Scale Feature Fusion (CSFF) module based on dual-channel pooling is designed to ensure the continuity of defect features, thereby resolving the semantic misalignment issue in traditional fusion. Third, a Multi-Kernel Strip Shuffle (MKSS) module is incorporated, utilizing decomposed convolution kernels to capture the geometric features of slender scratches. Finally, a knowledge distillation strategy is employed to transfer structured knowledge from a complex teacher model to a lightweight student model. Experiments on the Tianchi aluminum defect dataset demonstrate that WMC-DFINE achieves a mAP of 82.1%, which surpasses algorithms including YOLOv12, RT-DETR, and the baseline model DFINE. Furthermore, the distilled student model, WMC-DFINE-distill, improves the mAP by 3.2% compared to DFINE, reduces parameter count by 47%, and achieves an inference speed of 59.75 FPS on the experimental equipment. The proposed method effectively resolves the problem of balancing background suppression and defect detail feature preservation, offering a practical and efficient scheme for real-time industrial defect inspection.

## 1. Introduction

Owing to their light weight, excellent corrosion resistance, and high strength, aluminum profiles have been extensively applied in advanced industrial fields, including aerospace, rail transportation, automobile production, and precision electronics [1]. However, during manufacturing processes such as rolling, extrusion, and surface treatment, these profiles are highly susceptible to surface defects, such as scratches and dirty points, which are induced by complex processing environments and mechanical friction. Such defects not only degrade the esthetic appearance of products but also potentially trigger fatigue fractures in materials [2]. Traditional manual quality inspection suffers from inherent limitations, including profound subjectivity, susceptibility to visual fatigue, elevated missed detection rates, and overall low efficiency [3]. Consequently, automated inspection technologies driven by deep learning have progressively superseded manual inspection and have emerged as the mainstream paradigm in the industry. These automated systems primarily utilize advanced machine vision sensors to capture high-resolution images of the profile surfaces. However, processing such complex sensor data under varying industrial environments in real-time remains a significant challenge.

In recent years, detectors based on Convolutional Neural Networks (CNN) [4] have been highly prevalent, and can be generally divided into two-stage and single-stage structures. Exemplified by Faster R-CNN [5], two-stage methods first produce candidate bounding boxes via a Region Proposal Network (RPN) before performing classification and bounding box regression. However, the inherent inference latency caused by the two-stage processing paradigm makes it challenging to satisfy the stringent requirements for high-speed online inspection in aluminum profile production lines. Subsequently, the YOLO (You Only Look Once) [6] series has been extensively adopted owing to its superior inference speed. By incorporating Non-Maximum Suppression (NMS)-free strategies [7], efficient feature fusion [8], and regional attention mechanisms [9], these models significantly reduce post-processing latency and enhance global perception. In the context of defect detection, Li et al. [10] designed a weighted information representation module in the frequency domain tailored for wood surface defects. This module decouples low-contrast defects from the background and applies Gaussian filtering to eliminate noise and blur. Tang et al. [11] proposed a lightweight network integrating attention mechanisms and depthwise separable convolutions, thereby simultaneously improving the accuracy and speed of aluminum defect detection. Han et al. [12] reconstructed the network’s feature extraction layer by introducing the ALGC3 module combined with Ghost convolution; supplemented by dataset expansion and augmentation strategies, they achieved high-precision and low-complexity detection of aluminum alloy surface defects. Yin et al. [13] developed a lightweight BBW YOLO model, which incorporates BiFPN for enhanced feature fusion, utilizes the BiFormer dynamic sparse attention mechanism to extract critical features, and adopts WIoU v3 to optimize the loss function, thereby realizing high-precision and real-time detection of aluminum profile surface defects. Furthermore, Sun et al. [14] presented MCH-YOLOv12, which uses a multi-scale feature mining network to address the limitations of single-scale convolutions. This approach enhances feature expression and improves the identification of irregular defects. Despite the performance improvements achieved by advanced CNN-based detectors, their convolution kernels remain intrinsically constrained by local receptive fields, typically extracting only shallow textural features within the spatial domain. Consequently, these models are highly prone to conflating the inherent processing textures of aluminum surfaces with authentic subtle defects. Furthermore, for micro-defects such as dirty points, continuous downsampling steps in deep CNNs frequently result in feature loss.

As deep learning techniques have advanced rapidly, transformer-based object detection architectures have exhibited tremendous potential, surpassing CNNs in tackling complex metal surface defect detection tasks. Unlike CNNs, which rely on the mechanism of progressively aggregating features through local receptive fields, Transformer models efficiently capture long-distance interdependencies within image data via the self-attention operation. This paradigm exhibits significant advantages when processing metal surface defects characterized by large spans, non-rigid, or highly irregular morphologies. In 2020, Carion et al. [15] proposed DETR, which pioneered the remodeling of the object detection task as a set prediction problem. This study eliminated the anchor box design and the NMS post-processing steps inherent in traditional detectors, thereby realizing genuine end-to-end detection. However, the original DETR suffered from slow convergence rates and suboptimal detection accuracy for small objects, thereby severely restricting its practical application in industrial quality inspection. To address these limitations, Zhu et al. [16] subsequently proposed Deformable DETR, which introduced a multi-scale deformable attention module. By exclusively attending to sparse sampling locations around reference points rather than computing full-image attention, this design delivers a substantial cut in computing complexity, and concurrently effectively curbs the fading of tiny target features within deep network architectures. Building upon these foundations, Lv et al. [17] proposed Real-Time DETR (RT-DETR), designing an efficient hybrid encoder that enabled Transformer-based detectors to achieve real-time inference speeds for the first time, while simultaneously outperforming contemporaneous YOLO models in accuracy. In the specific domain of defect detection, Li et al. [18] integrated lightweight convolutions into RT-DETR to reduce the parameter count and introduced the DRBC3 module alongside deformable attention to enhance the neck network, successfully overcoming the dual challenges of limited aluminum defect samples and heavy computational complexity. Furthermore, SH-DETR, proposed by Wu et al. [19], incorporated channel random encoding and a parameter-free attention module, substantially improving the detection accuracy of steel surface defects while maintaining low model complexity. Similarly, Zuo et al. [20] put forward the Pavement-DETR detector, which incorporated the Conv3XC architecture to boost multi-level feature aggregation and designed a composite loss function integrating PIoU v2 and NWD.

Among the plethora of Transformer-based end-to-end detectors, Deformable Fine-grained DETR (DFINE), proposed by Peng et al. [21], stands as one of the most representative and cutting-edge algorithms in contemporary real-time object detection. This study innovatively highlights the inherent limitations of modeling bounding boxes based on the Dirac delta distribution, particularly when managing localization uncertainty. To address this, DFINE introduces the Fine-grained Distribution Refinement (FDR) module, which reconstructs the regression task into a coarse-to-fine iterative optimization process of probability distributions. This mechanism enables the model to achieve highly precise localization for defects with blurred boundaries or complex morphologies, significantly outperforming contemporaneous SOTA models such as RT-DETR on the COCO dataset. Nevertheless, despite its superior performance in general scenarios, DFINE exhibits notable limitations when directly applied to the complex inspection of aluminum surfaces. Specifically, although the model elegantly optimizes the regression distribution, its feature fusion remains strictly confined to the spatial domain. It lacks the capacity for proactive decoupling and filtering of intense aluminum background textures and frequency-band interferences, rendering it exceptionally difficult to isolate weak defect signals from complex background noise. Furthermore, existing feature extraction mechanisms struggle to maintain the structural continuity of large-span features, frequently causing long scratches to be erroneously detected as multiple fragmented segments.

To this end, this study addresses the critical challenges of intense background texture interference and the profound difficulty in detecting defects with extreme aspect ratios by proposing WMC-DFINE, a novel end-to-end defect detection model driven by frequency-domain perception and multi-scale feature enhancement. Building upon DFINE-s as the baseline model, WMC-DFINE is meticulously architected to deliver a high-precision detector with exceptional noise resistance. Furthermore, to achieve an optimal trade-off between detection accuracy and computational efficiency, we implement lightweight operations via model compression and knowledge distillation. The principal contributions of this study are summarized as follows:

1. Introduction of the Wavelet-Integrated Frequency Attention module: To address the prevalent issue of false positive detections induced by high-frequency textures, such as Orange Peel and Non-conductive on aluminum surfaces, we introduce the WIFA module based on the Haar wavelet transform. By decoupling features into the frequency domain, WIFA utilizes a gating mechanism to dynamically suppress high-frequency components representing background noise while enhancing the low-frequency semantic responses of authentic defects. This mechanism markedly enhances the model’s resilience and anti-disturbance capacity under conditions of intricate lighting and strong texture interference.
2. Incorporating the Multi-Kernel Strip Shuffle module: To address micro-defects and defects with extreme aspect ratios, the MKSS module is incorporated to inject geometric priors of localized and slender regions into the Transformer architecture via multi-branch convolution kernels. This design prevents the spatial dispersion of micro-defect features during global attention computations and preserves the structural continuity of slender defects, ultimately improving the detection of multi-morphological targets.
3. Design of the Cross-Scale Feature Fusion module: To mitigate the severe feature aliasing issues caused by conventional element-wise addition or channel concatenation when aggregating deep and shallow features, the CSFF module is designed with dual-channel pooling and spatial refinement units. Through cross-level channel interactions and subsequent post-fusion spatial refinement, CSFF achieves precise alignment and synergistic enhancement of deep semantic and shallow structural features.
4. Formulation of a Lightweight Scheme via Model Compression and Channel Distillation: We propose a highly efficient lightweight strategy that combines proportional channel compression with Channel-wise Distillation (CWD). This process compels a highly compact student model—comprising a mere 5.32 M parameters—to faithfully mimic the feature responses and prediction distributions of a high-precision teacher model. Experimental results reveal that this knowledge-distilled compact model not only markedly cuts down computational overhead but also achieves superior detection accuracy over the original baseline model.

## 2. Methodology

### 2.1. Wavelet-Integrated Frequency Attention Module

**Figure 1:**
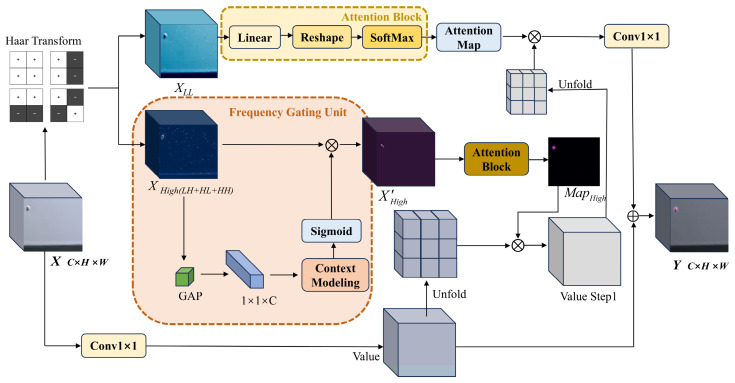
The detailed architecture of the proposed Wavelet-Integrated Frequency Attention module.

Within the domain of aluminum surface defect inspection, a primary challenge is that defect targets, such as Dirty Points and Paint Bubbles captured by optical sensors, inherently exhibit minuscule dimensions and low contrast. Furthermore, the inherent background textures of the aluminum substrate often cause frequency-domain aliasing with defect signals in the high-frequency spectrum. Inspired by Li et al. [22], who effectively employed wavelet transforms for lossless feature decomposition and enhancement in image super-resolution tasks, we propose the Wavelet-Integrated Frequency Attention (WIFA) module. The comprehensive architecture of this module is illustrated in Figure 1.

To achieve effective decoupling of background and defect features within the feature space, the module incorporates the Discrete Wavelet Transform (DWT) [23]. In industrial defect detection tasks, models exhibit profound sensitivity to the spatial resolution of features, and standard downsampling operations tend to result in the degradation of fine-grained feature information from micro-defects during hierarchical feature transmission. Consequently, we employ the Undecimated Discrete Wavelet Transform (UDWT) [24], which is essentially a wavelet transform operating with a stride of 1, to rigorously maintain the spatial resolution and dimensions of the feature maps throughout the multi-scale feature decomposition process. Formally, consider an input feature tensor $X\in R^{C\times H\times W}$, we utilize the Haar wavelet basis to construct the filters. Specifically, the four filter kernels with fixed weights, denoted as $K_{LL},K_{LH},K_{HL},K_{HH}$, are defined as in Equation (1).

(1) $$K_{LL}=\frac{1}{4}\left[\begin{array}{cc} 1 & 1\\ 1 & 1 \end{array}\right]\;\;\;\;\;K_{LH}=\frac{1}{4}\left[\begin{array}{cc} 1 & -1\\ 1 & -1 \end{array}\right]\;\;\;\;K_{HL}=\frac{1}{4}\left[\begin{array}{cc} 1 & 1\\ -1 & -1 \end{array}\right]\;\;\;\;K_{HH}=\frac{1}{4}\left[\begin{array}{cc} 1 & -1\\ -1 & 1 \end{array}\right]$$

Here, $K_{LL}$ computes the local average, while $K_{LH},K_{HL},K_{HH}$ capture the high-frequency components in the horizontal, vertical, and diagonal orientations, respectively. The feature decomposition process is mathematically formulated as in Equations (2) and (3).

(2) $$X_{LL}={X^{*}}_{s=1}K_{LL}$$

(3) $$X_{High}=\mathrm{Concat}\left({X^{*}}_{s=1}K_{LH},\;{X^{*}}_{s=1}K_{HL},\;{X^{*}}_{s=1}K_{HH}\right)$$

where ^*^_s=1_ denotes the convolutional operator with a stride size of 1. $X_{LL}$ captures the illumination distribution and overall structure of the image, while $X_{High}$ compactly encodes the texture variations and edge information. This operation furnishes comprehensive frequency perspectives for subsequent feature enhancement.

In the context of aluminum surface defect detection, relying solely on frequency separation methods is insufficient to address practical challenges. Owing to the significant disparities in the distribution characteristics between background noise and micro-defects across frequency channels, background textures typically manifest as global low-amplitude responses, whereas defects emerge as localized impulse responses. To achieve the precise isolation of the two, we design an adaptive Frequency Gating Unit (FGU). As a dynamic spectral filter, the core objective of this unit is to calibrate the weights of the high-frequency feature channels. This mechanism suppresses channels dominated by background textures and activates critical channels containing valid defect information. Initially, we compress the spatial dimensions of the high-frequency feature $X_{High}$ via Global Average Pooling (GAP) to derive a channel descriptor z, which characterizes the global texture distribution, as formulated in Equation (4).

(4) $$z_{k}=\frac{1}{H\times W}\sum_{i=1}^{H}\sum_{j=1}^{W}X_{High}(k,i,j)$$

Subsequently, we employed a 1D convolution to facilitate cross-channel interactions. The gating weights g and the refined high-frequency features $X_{High}^{\prime }$ are thereby generated as formulated in Equations (5) and (6).

(5) $$g=\sigma ({\mathrm{Conv}1D}_{k}(z))$$

(6) $$X_{High}^{\prime }=g⊙X_{High}$$

Here, σ(⋅) denotes the Sigmoid activation function, which maps the weights into (0,1). Ultimately, the refined high-frequency features $X_{High}^{\prime }$ are obtained via channel-wise multiplication.

After obtaining the processed frequency-domain features, the module abstains from directly superimposing them linearly onto the original features. Instead, they are routed through two sequential Predicted Window Attention Blocks. Initially, the input feature tensor X is mapped into a value stream V via linear projection. The first-stage attention mechanism is primarily designed to mitigate the issue of missed detections. Guided by the purified high-frequency features $X_{High}^{\prime }$, it employs a highly efficient predictive mechanism to directly forecast the attention distribution within local windows from the high-frequency textures. Specifically, the network predicts the high-frequency attention map $Map_{High}$ via a linear layer, which is subsequently normalized within the local spatial window using the Softmax function. Subsequently, local matrix multiplication is utilized to apply this attention map to the value stream V, yielding the intermediate feature $V_{step1}$, as formulated in Equations (7) and (8).

(7) $$Map_{High}=\mathrm{Softmax}\left(W_{High}\cdot \mathrm{Pool}(X_{High}^{\prime })\right)$$

(8) $$V_{step1}=Map_{High}⊗V$$

Here, $W_{High}$ represents the linear projection matrix. Subsequently, $V_{step1}$ is propagated to the second-stage attention block, utilizing the low-frequency component $X_{LL}$ as the guiding signal. Given that $X_{LL}$ encapsulates the illumination variations and macroscopic structural information of the image, the corresponding low-frequency attention map $Map_{Low}$ derived from it is employed for feature smoothing and contextual unification. Finally, the processed feature stream $V_{out}$ is routed through an output projection layer and subjected to a residual link with the initial input tensor X. This yields the final output of the WIFA module, as mathematically formulated in Equations (9) and (10).

(9) $$Map_{Low}=\mathrm{Softmax}\left(W_{Low}\cdot \mathrm{Pool}(X_{LL})\right)$$

(10) $$V_{out}=Map_{Low}⊗V_{step1}$$

Through such profound frequency-domain interactions, the WIFA module not only bolsters the capability to decouple background noise but also significantly enhances the robustness of the model in complex industrial environments.

### 2.2. Multi-Kernel Strip-Shuffle Model

**Figure 2:**
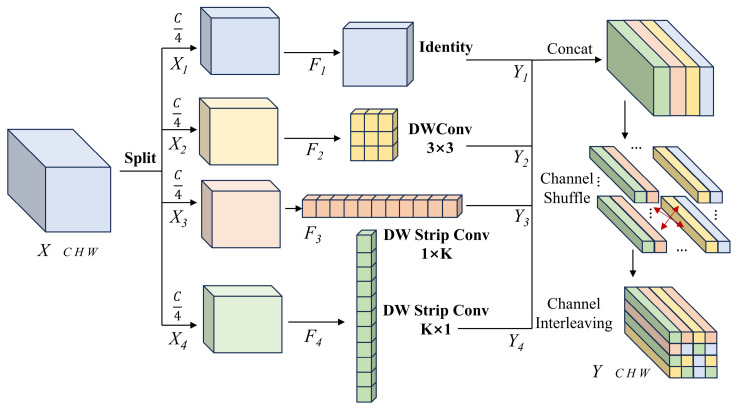
The detailed architecture of the Multi-Kernel Strip Shuffle module.

In complex scenarios involving aluminum surface defect detection, morphological disparities among the defect targets are highly pronounced. In addition to typical micro-defects such as Paint Bubbles and Dirty Point, the detection dataset encompasses a substantial proportion of defects with extreme aspect ratios, such as Slender Scratches and Jet Flow trajectories. Despite the inherent strength of the Transformer architecture in capturing global long-range dependencies through self-attention mechanisms, it has notable limitations when processing defects characterized by these specialized morphologies. Specifically, standard global attention mechanisms are highly prone to overlooking micro-defects, thereby resulting in severe missed detections of small targets. Furthermore, Transformers conventionally employ fixed-shape isotropic attention windows that struggle to align precisely with the geometric distributions of Slender Scratches. This isotropic constraint frequently causes slender defects to be truncated or suffer from severe feature discontinuities. To explicitly address this critical issue, drawing inspiration from the InceptionNeXt philosophy introduced by Yu et al. [25] in high-efficiency CNN design, we incorporated the Multi-Kernel Strip Shuffle (MKSS) module. This module decomposes computationally intensive large-kernel convolutions into multiple direction-specific branches. By employing strip convolutions to independently capture contextual information along the horizontal and vertical axes, MKSS constructs a dynamic receptive field capable of adaptively matching diverse defect morphologies at a remarkably low computational cost. The detailed architecture of the MKSS module is illustrated in Figure 2.

In the MKSS module, let X denote the input feature tensor. The module initially employs a channel grouping strategy, uniformly partitioning X into four sub-groups along the channel dimension. Distinct convolutional operations are subsequently applied to each subgroup. We define a set of geometric operators, denoted as $\Phi =\{F_{1},F_{2},F_{3},F_{4}\}$, which correspond to the identity mapping branch, the local context branch, the horizontal strip branch, and the vertical strip branch, respectively. The specific transformation process is mathematically formulated as in Equations (11) and (12).

(11) $$Y_{1}=F_{1}(X_{1})=X_{1}$$

(12) $$Y_{2}=F_{2}(X_{2})=W_{3\times 3}*X_{2}$$

Here, ∗ denotes the depthwise separable convolution operation. The identity mapping branch abstains from any convolutional operations; its principal objective is to rigorously preserve the original distribution of the input features. This design guarantees the lossless propagation of gradients during backpropagation within the deep network, thereby preventing the loss of fine-grained textures on the aluminum surface under complex feature transformations. The local context branch employs a standard $W_{3\times 3}$ convolutional kernel to capture locally compact geometric features. This branch is specifically tailored for ordinary, regular defects with aspect ratios approximating 1:1. To explicitly address slender defects, the MKSS module incorporates two orthogonal strip convolution branches, as mathematically formulated in Equations (13) and (14).

(13) $$Y_{3}=F_{3}(X_{3})=W_{1\times K}*X_{3}$$

(14) $$Y_{4}=F_{4}(X_{4})=W_{K\times 1}*X_{4}$$

Here, the length of the strip kernel was set to 11. The $F_{3}$ branch constructs an elongated horizontal receptive field, capable of precisely enveloping horizontal Scratches while excluding vertical background interference. Similarly, the $F_{4}$ branch focuses on slender features along the vertical axis, thereby enabling the network to capture ultra-long-range spatial correlations at an extremely low parameter cost.

Following the multi-scale feature decomposition procedure, the outputs generated by each individual branch are concatenated across the channel dimension. Owing to the inherent channel independence of depthwise convolutions, the horizontal and vertical features remain strictly isolated across channels. Consequently, it is challenging for the network to directly leverage them for representing targets with complex, multidirectional geometric characteristics. To dismantle this channel isolation and facilitate cross-channel information interaction, a Channel Shuffle operation is integrated into the MKSS module. By structurally rearranging the channels, Channel Shuffle compels the features from disparate groups to uniformly interweave along the channel dimension. In the resultant output, adjacent channels originate from distinct geometric branches. This configuration allows subsequent network layers to adaptively fuse these multidirectional features by learning inter-channel weights. Consequently, the network synthesizes equivalent receptive fields across diverse orientations, significantly improving its ability to recognize defects with extreme aspect ratios.

While relentlessly pursuing high detection accuracy, MKSS adheres to the principles of real-time processing by maintaining a lightweight architecture. We systematically compared the theoretical computational complexity of MKSS with that of standard large-kernel convolutions. Suppose the input and output channel counts are both *C*, with the spatial size of the feature map being *H* × *W.* If a standard *K* × *K* large-kernel depthwise convolution is employed to attain an equivalent receptive field, its FLOPs can be formulated as in Equation (15):

(15) $$\Omega \approx H\cdot W\cdot C\cdot K^{2}$$

Within the MKSS module, excluding the identity mapping and standard small-kernel convolution, the predominant computational complexity is concentrated within the two orthogonal strip branches, with each branch processing only one-quarter of the input channels. By disregarding the bias terms, its corresponding computational volume is mathematically formulated as in Equation (16).

(16) $$\Omega_{mkss}\approx H\cdot W\cdot \left(\frac{C}{4}\cdot 3^{2}+\frac{C}{4}\cdot (1\cdot K)+\frac{C}{4}\cdot (K\cdot 1)\right)\approx H\cdot W\cdot \frac{C}{2}\cdot K$$

By juxtaposing Equations (15) and (16), it is evident that MKSS significantly reduces the computational complexity from a quadratic dependency on the kernel size K to a linear scale. Theoretically, MKSS substantially reduces computational complexity while meticulously preserving the representational capacity for defects with complex morphologies through its multi-kernel shuffle mechanism. Consequently, the MKSS module effectively rectifies the prevailing deficiencies in aluminum surface defect detection when addressing defects with extreme aspect ratios, while concurrently guaranteeing real-time inference speeds suitable for industrial automated inspection.

### 2.3. Cross-Scale Feature Fusion Module

**Figure 3:**
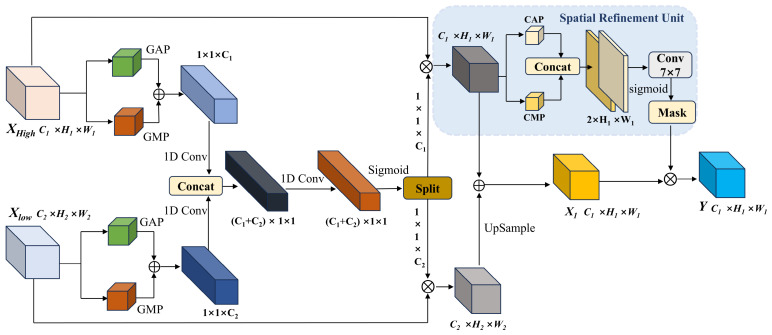
Detailed architecture of the Cross-Scale Feature Fusion module.

Within deep neural networks, shallow features preserve the intricate edge and textural details of aluminum defects; however, they inherently lack the discriminative capacity required for accurate defect categorization. Conversely, deep features encapsulate highly abstract semantic information but suffer from severely compromised localization precision owing to successive downsampling operations. Conventional fusion paradigms typically rely on naive element-wise addition or linear concatenation. Such fusion methodologies critically disregard the representational discrepancies inherent in multiscale features when depicting defects. Consequently, they frequently cause profound deep semantic information to overshadow faint shallow details, or induce severe feature aliasing during the upsampling phase. To explicitly circumvent these bottlenecks, we introduce the Cross-Scale Feature Fusion (CSFF) module. Based on dual-channel pooling, CSFF facilitates cross-level channel interactions and post-fusion spatial refinement. This synergistic mechanism guarantees the precise alignment and robust enhancement of both deep and shallow features. The structural design of the CSFF module is depicted in Figure 3.

To accurately ascertain which features are pivotal along the channel dimension, the construction of a robust channel descriptor is imperative. Conventional Global Average Pooling (GAP) exhibits an inherent tendency to smooth the feature distribution; although this characteristic is advantageous for suppressing background noise, it is highly susceptible to neglecting critical small-scale features. Consequently, the CSFF module incorporates a dual-channel pooling strategy, leverages both average and max pooling mechanisms to meticulously preserve the richness of the feature representations. Let the input shallow high-resolution feature be denoted as $X_{high}$, and the deep low-resolution feature be denoted as $X_{low}$. Within the CSFF module, the global average and global maximum of the respective feature maps are computed and subsequently aggregated via element-wise addition to derive the channel descriptor z, as mathematically formulated in Equation (17).

(17) $$z_{k}=\mathrm{GAP}(X_{k})+\mathrm{GMP}(X_{k}),\;\;k\in \{high,low\}$$

Here, GAP predominantly captures background contextual information, whereas GMP focuses on extracting salient target regions. Upon acquiring the hierarchical descriptors $z_{high}$ and $z_{low}$ from the deep and shallow layers, the module abstains from computing their channel weights independently; instead, a synergistic combinatorial paradigm is adopted. Specifically, CSFF concatenates the descriptors from both scales along the channel dimension, synthesizing a joint feature vector that comprehensively encapsulates the multi-scale context. Subsequently, a 1D convolutional layer with shared weights is deployed to perceive cross-channel local dependencies, thereby generating adaptive fusion weights, as formulated in Equation (18).

(18) $$w_{joint}=\sigma \left({\mathrm{Conv}1D}_{k}\left(\left[z_{high},z_{low}\right]\right)\right)\;\;\;\;\left[w_{high},w_{low}]=\mathrm{Split}(w_{joint}\right)$$

Here, [] denotes the concatenation operation, σ represents the Sigmoid activation function, and the Split operation partitions the generated weights into two distinct segments. This mechanism enables the network to dynamically ascertain whether the current scene relies more heavily on fine-grained details or high-level semantics. Ultimately, the original features are recalibrated using these generated weights; subsequently, the deep features are upsampled and fused with the shallow features, as mathematically formulated in Equation (19).

(19) $$X_{1}=X_{high}⊙w_{high}+\mathrm{Up}(X_{low}⊙w_{low})$$

where ⊙ denotes channel-wise broadcasting multiplication, and Up represents the upsampling operation. Feature maps subjected to channel-wise weighting are highly susceptible to aliasing at object boundaries, which inevitably blurs the target edges. To further elevate localization precision, a Spatial Refinement Unit is incorporated at the terminus of the module, dedicating its focus primarily to the spatial positioning of the targets. We independently extract the spatial average map and the spatial maximum map of the fused features along the channel axis, thereby compressing the features into spatial statistical representations. Subsequently, a convolutional layer with a large receptive field is employed to generate the spatial mask $M_{s}$, as formulated in Equation (20).

(20) $$M_{s}=\sigma \left({\mathrm{Conv}}_{7\times 7}\left(\left[{\mathrm{Mean}}_{c}(X_{1}),{\mathrm{Max}}_{c}(X_{1})\right]\right)\right)$$

The output features are subsequently filtered through $M_{s}$. This process suppresses cluttered features introduced during fusion, ensuring the detection head receives feature maps with enriched multiscale semantics and precise spatial boundaries. This ultimately improves overall detection performance.

### 2.4. Overall Network Architecture of WMC-DFINE

**Figure 4:**
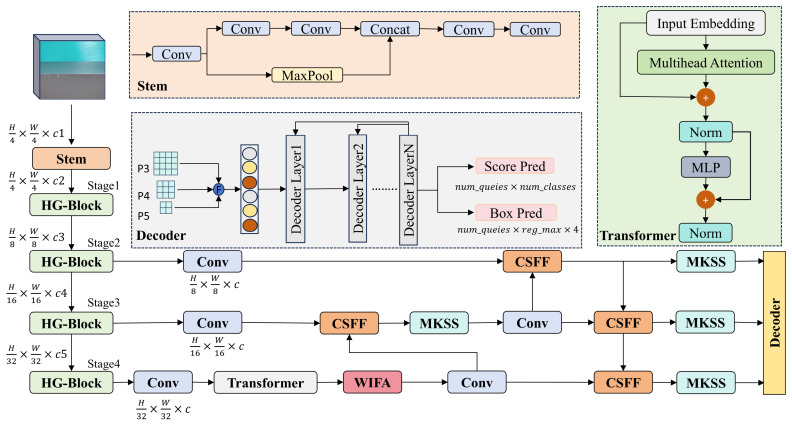
The overall network architecture of the proposed WMC-DFINE model.

The preceding sections have comprehensively elaborated on the three core modules: the Wavelet-Integrated Frequency Attention, the Multi-Kernel Strip Shuffle, and the Cross-Scale Feature Fusion. The overall network architecture of the proposed WMC-DFINE is illustrated in Figure 4.

Built upon the HGNetv2 backbone and centered around an enhanced hybrid encoder, the proposed WMC-DFINE systematically addresses the inherent challenges of aluminum surface defect detection from three distinct paradigms: frequency-domain denoising, feature alignment, and morphological perception. Initially, an input image is fed into the HGNetv2 backbone network for hierarchical feature extraction. The output feature maps from the final three stages, denoted as P3, P4, and P5, serve as the foundational representations for subsequent processing. Specifically, P3 preserves intricate spatial geometric details, thereby facilitating the detection of micro-defects; conversely, P5 encapsulates profound high-level semantic information, which is pivotal for identifying defect categories. Given that the deep-level feature P5 is highly susceptible to interference from inherent aluminum surface textures, we strategically incorporate the WIFA module subsequent to the Transformer-based global context modeling. This module dynamically suppresses background noise components within the frequency-domain space. During the feature fusion phase, the CSFF module employs dual-channel pooling to compel multi-scale features to interact along the channel dimension. This mechanism effectively overcomes the semantic misalignment induced by conventional linear concatenation, ensuring the structural continuity and completeness of defects across hierarchical feature maps. Following the output from the CSFF module, we deploy the MKSS module to replace the original RepNCSPELAN architecture. Leveraging its multi-branch anisotropic convolution kernels and small convolution kernels, MKSS empowers the network to adaptively focus on the specialized morphologies of micro point-like and slender strip-like defects.

### 2.5. Model Lightweighting and Channel-Wise Knowledge Distillation

**Figure 5:**
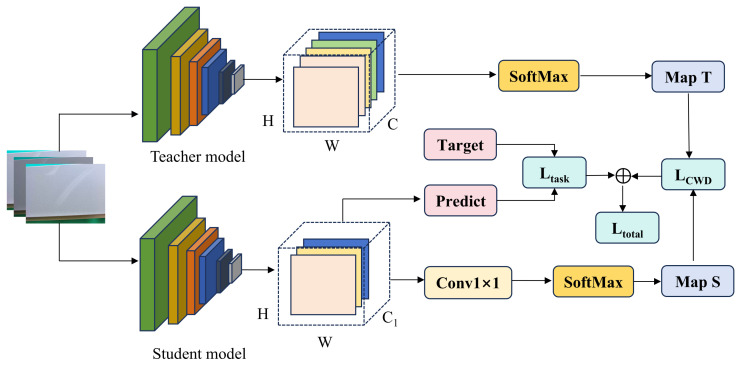
The pipeline of the Channel-wise Distillation strategy adopted in our framework.

To satisfy the stringent demands for real-time inference in industrial deployment scenarios, we propose a lightweight variant of the model and incorporated a Knowledge Distillation (KD) strategy to robustly preserve detection accuracy. In the context of prevalent aluminum surface defects, the majority of anomalous targets inherently manifest as sparse salient regions dominated by pervasive background textures. Consequently, conventional pixel-wise distillation often yields suboptimal performance by over-constraining the alignment of redundant background noise. To circumvent this limitation, we construct a highly compact student network and adopt the Channel-wise Distillation (CWD) [26] methodology. By strictly aligning the probability distributions within the feature channels, CWD explicitly guides the student model to faithfully mimic the teacher model’s capacity for extracting multi-scale geometric features. This paradigm ultimately yields an exceptionally efficient lightweight model. The specific distillation pipeline is illustrated in Figure 5.

We adopt width scaling [27] to construct a lightweight student network. Specifically, while preserving the overall network architecture, we apply a uniform width multiplier to scale the channels across every convolutional layer of the teacher network. Given that the parameter count of a convolutional layer is quadratically proportional to the number of channels, this width multiplier substantially compresses both the parameter volume and the theoretical computational complexity of the student model, thereby significantly elevating the inference speed. The student and teacher networks maintain an identical architectural layout. This structural consistency eradicates interference stemming from feature misalignment, enabling the distillation process to focus exclusively on learning feature representations. Within the deep convolutional network, to maximize the student network’s acquisition of the teacher’s feature extraction capabilities, we strategically establish the distillation alignment points at the critical nodes of the feature pyramid—the output terminals of the MKSS module. Furthermore, we adopted the CWD strategy to transform the activation maps into probability distributions. Let $F^{T},F^{S}$ denote the feature tensors of the teacher and student networks at the same hierarchical level, respectively. For the activation map of the c-th channel, we utilize the Softmax function to perform spatial normalization, effectively converting it into a channel-wise probability distribution, as formulated in Equation (21).

(21) $$\phi (y_{c,i})=\frac{\exp(\frac{y_{c,i}}{t})}{\sum_{j=1}^{H\cdot W}\exp(\frac{y_{c,j}}{t})}$$

Here, i denotes the spatial position index, and *t* represents the temperature coefficient. Through this transformation, $\phi (y_{c})$ effectively mitigates the numerical scale mismatch induced by the disparity in channel dimensions between the teacher and student networks, inherently shifting the focus toward the spatial localizations of the defects. Subsequently, we employ the Kullback–Leibler (KL) divergence to quantify the discrepancy between their respective channel distributions. The distillation loss function, denoted as $L_{CWD}$, is mathematically defined as in Equation (22).

(22) $$L_{CWD}(F^{T},F^{S})=\frac{t^{2}}{C}\sum_{c=1}^{C}\sum_{i=1}^{H\cdot W}\phi (y_{c,i}^{T})\cdot \log\left(\frac{\phi (y_{c,i}^{T})}{\phi (y_{c,i}^{S})}\right)$$

By capitalizing on the asymmetric nature of the KL divergence, this loss function adaptively intensifies the constraints imposed upon the foreground defect regions. Specifically, when the teacher network detects a salient anomaly at a given location, it compels the student network to rigorously learn the strip-like and point-like features extracted by the MKSS module; conversely, the constraints applied to the background regions remain relatively lenient. Ultimately, the overall training loss of the network is formulated as a weighted sum of the detection and distillation losses, as shown in Equation (23).

(23) $$L_{total}=L_{detect}+\lambda \cdot L_{CWD}$$

Here, λ denotes the balancing coefficient. Driven by this strategic distillation paradigm, the student network comprehensively assimilates the teacher network’s representational capacity for complex geometric defects, whilst sustaining an exceptionally lightweight architecture.

## 3. Results and Analysis

### 3.1. Tianchi Aluminum Profile Surface Defect Dataset

**Figure 6:**
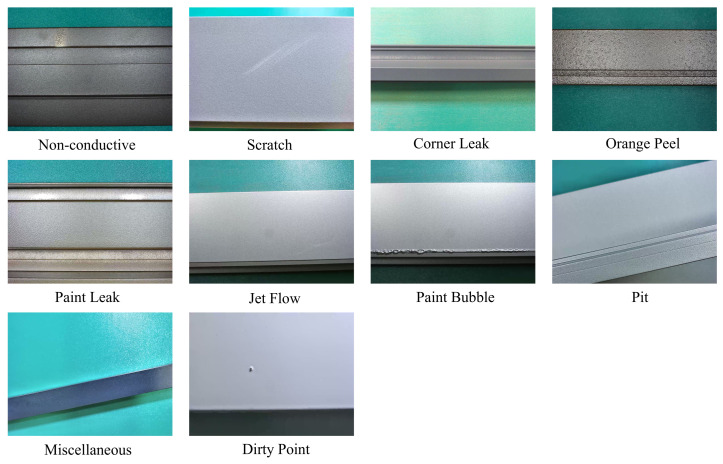
Representative examples of the ten distinct defect categories within the aluminum surface defect dataset.

For our experimental evaluation, we adopted the public dataset released by Alibaba Tianchi. Acquired directly from authentic industrial aluminum profile production lines, this dataset comprises high-resolution RGB images encompassing the ten most typical defect categories encountered during aluminum manufacturing. As enumerated, these ten distinct defect classes are: Non-conducting, Scratch, Corner Leak, Orange Peel, Paint Leak, Jet Flow, Paint Bubble, Pit, Miscellaneous, and Dirty Point. Typical defect samples included in the dataset are displayed in Figure 6.

**Figure 7:**
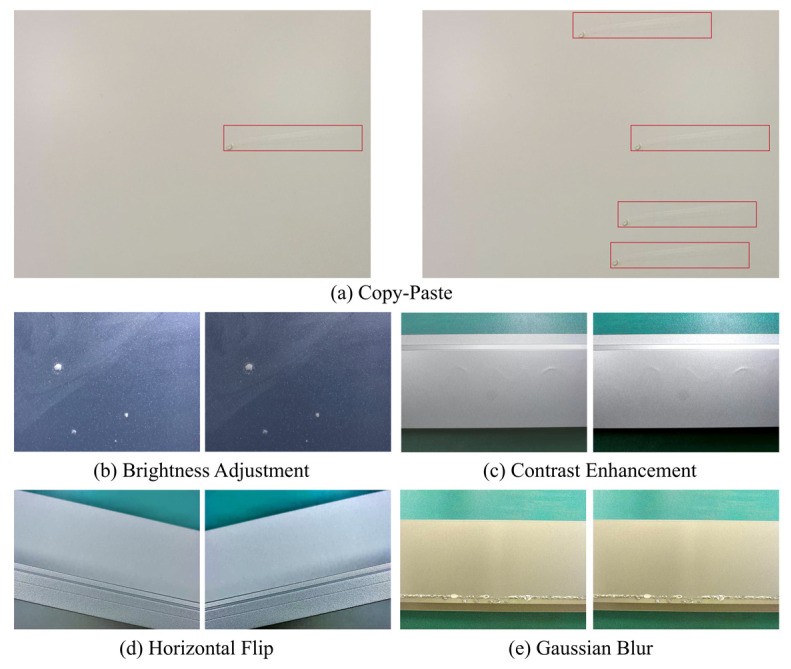
Visual illustrations of the five data augmentation techniques applied in this study.

In real-world scenarios of aluminum surface defect detection, inherent challenges persist, including complex illumination environments, motion blur, and underlying dataset constraints. To systematically boost the generalization ability and structural robustness of our model, we implement five distinct data augmentation techniques. The qualitative visualizations generated by these augmentation techniques are depicted in Figure 7.

To address the challenge of few-shot defects such as Paint Bubble and Jet Flow, we adopt the Copy–Paste [28] strategy, as illustrated in Figure 7a. Specifically, we construct a material library for these minority defect classes. We randomly select defect targets and paste them onto arbitrary defect-free background images. The Poisson blending algorithm is then applied to eliminate artifacts at the pasting boundaries seamlessly. By preserving the original background semantics, the Copy–Paste strategy augments the instance count of few-shot categories, effectively mitigating the adverse effects of long-tailed data distribution. Concurrently, to simulate diverse interferences in industrial imaging, we introduce four fundamental transformations. First, given the highly reflective nature of aluminum surfaces, we employ random brightness and contrast adjustments. This operation simulates diurnal lighting fluctuations on the production line, allowing the model to concentrate on the inherent texture features of defects, as shown in Figure 7b,c. Second, considering the random orientations of aluminum profiles on the conveyor belt, we apply horizontal flip augmentation. This geometric transformation ensures that detection performance is invariant to the placement direction of the material, as illustrated in Figure 7d. Finally, accounting for potential motion blur or camera defocus caused by high-speed conveyor operation, we incorporate random Gaussian blur during training. This prevents the network from overfitting to high-frequency noise, as depicted in Figure 7e.

**Figure 8:**
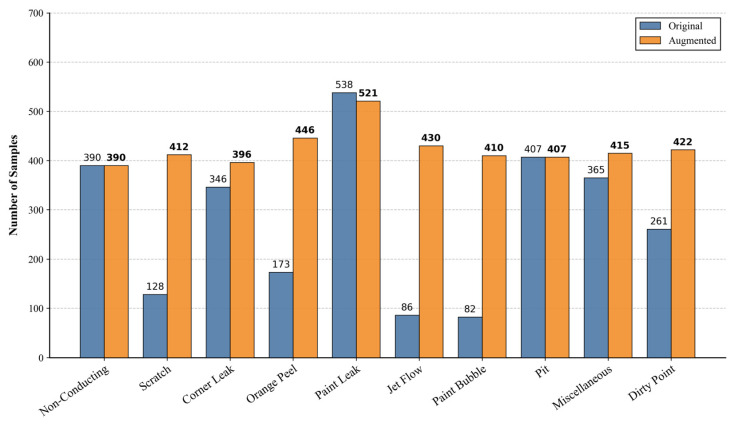
Class distribution of the aluminum surface defect dataset before and after data augmentation.

The original dataset contains 2776 defect images. After applying these five data augmentation techniques, we expand the total number of samples to 4249. The distribution of the augmented dataset is shown in Figure 8.

The rebalanced dataset is randomly partitioned into training, validation, and testing sets following a 7:2:1 ratio. The training set, comprising 2970 images, is utilized to optimize the network’s weights. The validation set of 850 images and the testing set of 429 images are respectively employed for quantitative performance benchmarking and qualitative evaluation.

### 3.2. Experimental Environment

To ensure the fairness and reproducibility of our evaluations, all experiments are conducted on a uniformly configured server. The detailed hardware and software specifications are summarized in Table 1.

### 3.3. Evaluation Metrics and Implementation Details

To comprehensively assess the efficacy of our proposed method for the aluminum surface defect detection task, this study employs Precision (P), Recall (R), Mean Average Precision (mAP), parameter count, and Frames Per Second (FPS) as the primary evaluation metrics.

Precision quantifies the percentage of true positive samples among all instances classified as positive by the model, while Recall represents the ratio of true positive detections to the total number of annotated ground-truth positives. Their formal definitions are given in Equation (24).

(24) $$P=\frac{TP}{TP+FP}\;\;\;R=\frac{TP}{TP+FN}$$

Here, TP (True Positive) denotes the number of accurately detected defects, FP (False Positive) represents the number of background instances erroneously identified as defects, and FN (False Negative) designates the number of undetected defects. Average Precision (AP) is defined as the area under the Precision–Recall (PR) curve, serving to quantify the detection performance for a singular category. Meanwhile, mAP is the mean of the AP values across all categories, acting as the core metric for assessing the overall average performance across all classes. The corresponding calculation formulas are expressed in Equation (25).

(25) $$AP=\int_{0}^{1}P(R)dR\;\;\;\;mAP=\frac{1}{C}\sum_{i=1}^{C}AP_{i}$$

In this context, C denotes the total number of defect categories, which is ten in this study. While Precision and Recall evaluate specific operating thresholds, they are mutually restrictive and cannot independently represent overall performance. Furthermore, standard accuracy is unsuitable for object detection due to the severe imbalance between foreground targets and background regions. Therefore, mAP is utilized as the primary precision metric, as it computes the area under the Precision–Recall curve, providing a comprehensive assessment that holistically balances both classification correctness and localization quality across all categories. Driven by the stringent real-time requirements of industrial deployment, and to rigorously validate the efficacy of channel compression and knowledge distillation, this study incorporates parameter count and Giga Floating-point Operations (GFLOPs) to evaluate the spatial complexity and computational complexity of the model. Furthermore, the FPS metric is utilized to gauge the actual inference speed of the model deployed on the RTX 4090D GPU.

To ensure a fair evaluation, we adopted the baseline’s AdamW optimizer [29], with the momentum parameters $\beta_{1},\beta_{2}$ set to 0.9 and 0.999, respectively, and the weight decay coefficient configured to 1 × 10^−4^. Its decoupled weight decay mechanism effectively mitigates overfitting, ensuring stable model convergence on aluminum profile datasets characterized by severe background noise and ambiguous defect boundaries. The model is trained for 132 epochs, and the batch size is set to 16. The detailed hyperparameter configurations are systematically outlined in Table 2.

**Figure 9:**
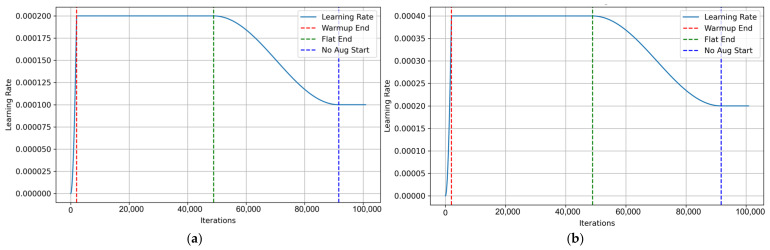
The dynamic learning rate scheduling curves utilized during the training process: (**a**) learning rate curve of the backbone network; (**b**) learning rate curve of the detection head and other auxiliary components.

To strike an optimal balance between the model’s convergence speed and training stability, we employ a dynamic learning rate scheduling strategy. Considering that the backbone network is initialized using pre-trained parameters while the detection head is initialized with random weights, we introduce a Differential Learning Rate (DLR) mechanism. Specifically, the initial learning rate for the backbone is set to 2 × 10^−4^, as depicted in Figure 9a, whereas the initial learning rate for the detection head and other auxiliary components is configured to 4 × 10^−4^, as illustrated in Figure 9b.

During the initial three training epochs, t the learning rate is linearly ramped up to its baseline value. This warmup procedure is devised to alleviate the problem of severe gradient vanishing. Subsequently, the learning rate is maintained at this maximum value for 64 epochs, ensuring sufficient extraction of image features. Following this plateau phase, the training transitions into a cosine annealing phase [30], where the learning rate decays smoothly along a cosine curve. In the final 12 epochs of training, to mitigate the data distribution shift introduced by Mosaic and Mixup, the learning rate is fixed at an extremely low value for fine-tuning. This final phase enhances the model’s generalization capability in real-world scenarios.

### 3.4. Channel Compression and Model Distillation

We comprehensively evaluate the performance of the proposed WMC-DFINE model, which seamlessly integrates the WIFA, MKSS, and CSFF modules. Compared with the baseline DFINE, WMC-DFINE elevates the mAP@0.5 from 76.3% to 82.1%, achieving an improvement of 5.8%. However, the increased computational complexity can become a bottleneck for concurrent multi-camera inspection in actual production lines. To satisfy the stringent real-time requirements of industrial automated inspection while preserving high detection accuracy, we introduce a lightweight variant denoted as WMC-DFINE-distill. This variant executes channel compression by incorporating a width multiplier w, and subsequently leverages knowledge distillation to compensate for any resultant degradation in accuracy. We configure four distinct experimental setups with w∈{1.0,0.75,0.50,0.25}. The corresponding empirical results are detailed in Table 3.

When w = 1.0, the mAP@0.5 peaks at 82.1%. Although this enhanced model attains the highest detection precision, its parameter count concurrently reaches 13.48 M. Conversely, at w = 0.25, the model yields the most lightweight architecture; however, the mAP precipitously drops to 75.5%, and the marginal gain in FPS is negligible compared to the w = 0.50 configuration. At w = 0.50, the model achieves the best trade-off between detection accuracy and computational efficiency. In particular, relative to the baseline with w = 1.0, its parameter count and computational complexity are substantially reduced by 60.5% and 59%. To rigorously evaluate real-time industrial deployment feasibility, we further analyzed the FPS under a strict latency-sensitive setup. Specifically, the evaluation utilized a batch size of 1 on an RTX 4090D workstation and included both the forward pass and NMS overheads. Under these stringent conditions, the w = 0.50 model accelerates to 59.82 FPS, which effectively doubles the standard real-time requirement of 30 FPS for industrial automated optical inspection (AOI) systems. Consequently, we definitively designate WMC-DFINE (w = 0.50) as the student model for the subsequent knowledge distillation pipeline.

To compensate for the accuracy degradation induced by channel compression, we implement a knowledge distillation strategy. The high-precision WMC-DFINE serves as the teacher model to rigorously guide the training of the lightweight student model, WMC-DFINE-distill (w = 0.50). Through thorough regularization of feature activations and soft-label distributions, the rich semantic knowledge contained in the teacher model is successfully transferred to the student network. The corresponding experimental findings are summarized in Table 4.

**Figure 10:**
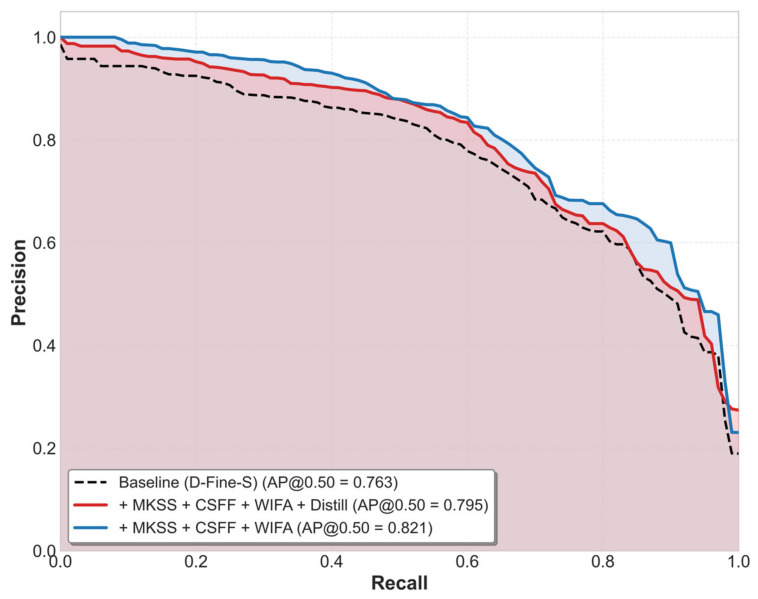
PR curves for the teacher model, the student model, and the baseline model.

Following the distillation process, the mAP@0.5 of the student model increases from 78.3% to 79.5%, achieving an absolute improvement of 1.2 percentage points. Compared with the original baseline model, the distilled WMC-DFINE-distill achieves a 3.2% increase in detection accuracy while simultaneously reducing the parameter count by 47%. Furthermore, it maintains an inference speed of approximately 59.75 FPS, ensuring that high precision does not compromise operational latency in industrial environments. The Precision–Recall (PR) curves for the teacher model, the student model, and the baseline model are illustrated in Figure 10.

The teacher model, WMC-DFINE, achieves the largest area under the curve, achieving an mAP@0.50 of 82.1%. Impressively, the lightweight student model robustly retains 96.8% of the teacher’s performance, reaching 79.5%. This demonstrates that distillation effectively transfers the teacher’s knowledge to the student, compensating for the performance drop caused by the reduced model scale. Despite a substantial reduction in the parameter count, the distilled student model outperforms the baseline by 3.2%, yielding an architecture that is simultaneously lighter and more accurate than the original baseline.

### 3.5. Structural Novelty Validation

To further rigorously justify the structural novelty of the proposed modules and their specific adaptation to the domain of aluminum profile surface defect detection, we conducted a set of targeted experiments. We systematically compared our customized modules against the classical attention mechanisms and efficient building blocks from which they draw mathematical inspiration.

As shown in Table 5, we compared our proposed modules against standard baseline mechanisms, including SE [31], InceptionNeXt, and CBAM [32], to rigorously validate their structural novelty and domain-specific necessity.

In comparing frequency decoupling and spatial attention, the standard SE module slightly improves Precision to 79.6% by reweighting spatial channels but fails to detect micro-defects masked by high-frequency background noise, yielding a low Recall of 72.3%. In contrast, WIFA module uses Discrete Wavelet Transform to decouple features into the frequency domain. By filtering background texture noise, WIFA trades a negligible Precision drop settling at 78.9% for a sharp Recall increase from the baseline 70.7% to 79.5%, proving frequency-domain denoising is essential for detecting missed defects on textured aluminum surfaces. For extreme aspect ratio perception, MKSS module and standard InceptionNeXt have similar parameter counts of approximately 10.3 M and comparable computational overheads, as both use large-kernel convolution decomposition. However, MKSS is specifically designed with structural re-parameterization and an explicit channel shuffle mechanism to preserve the continuous geometric priors of slender defects. This targeted adaptation allows MKSS to achieve 80.7% Precision and 77.0% Recall, which significantly outperforms the 80.1% Precision and 74.2% Recall produced by the standard InceptionNeXt block. This validates that our morphological adaptation prevents strip-like feature dispersion. Finally, comparing cross-scale and intra-scale enhancement demonstrates that CBAM improves Precision to 80.3% via intra-scale masking but fails to prevent small defect loss during upsampling due to poor cross-scale awareness, limiting its Recall to 73.5%. CSFF resolves this semantic misalignment via cross-level channel interaction before fusion; by seamlessly bridging high-level semantics and low-level details, it simultaneously boosts Precision to 80.7% and Recall to 77.1%.

These performance gaps confirm that the structural innovations of WMC-DFINE are indispensable, driving the overall mean average precision to 82.1%.

### 3.6. Ablation Study

To verify the individual effectiveness of the three core modules proposed in this study—namely WIFA, MKSS, and CSFF—and to further investigate their synergistic interactions, we design nine ablation experiments. The quantitative results are systematically detailed in Table 6.

To ensure the reliability of the experimental results, all configurations in this ablation study were evaluated across multiple independent runs. Adding the WIFA module alone to the baseline model elevates the mAP@0.5 to 78.2%, a 1.9 percentage point improvement. By leveraging DWT to decouple features into high- and low-frequency components, WIFA effectively suppresses reflective glare and texture noise on aluminum surfaces while accentuating the edge features of defects. Similarly, adding the MKSS module alone increases the mAP@0.5 to 77.9%, as its multiscale kernels enable the model to better capture defects with high aspect ratios. Furthermore, the CSFF module alone boosts the mAP@0.5 to 78.2%, a 1.9 percentage point improvement, by bridging the transmission barriers between deep semantic information and shallow detail information, thereby mitigating the information gap.

Among the dual-module configurations, the combination of MKSS and CSFF is the top performer, reaching an mAP@0.5 of 80.2%. MKSS expands the receptive field to capture rich multi-scale information, while CSFF facilitates the fusion of these features across different layers. These two modules exhibit structural complementarity as MKSS extracts diverse features, which are then efficiently integrated by CSFF. Thus, compared with using either module alone, their combination achieves an approximately 2.3% improvement. The combinations of WIFA and MKSS, and WIFA and CSFF also yield improvements, with mAP@0.5 reaching 79.1% and 78.9%, respectively, but the improvement is slightly lower than that of the MKSS and CSFF combination. WIFA focuses on frequency domain denoising. When it is paired with CSFF without MKSS, the network still lacks the critical capability to accurately extract the geometric features of defects with extreme aspect ratios, creating a feature extraction bottleneck that inherently limits overall performance. When the three proposed modules are combined together, our model reaches the best detection performance, with mAP@0.5 rising to 82.1% which is a 5.8 percentage point improvement over the baseline and a precision of 83.9%. Concurrently, the F1-Score significantly improves from 74.8% to 79.9%. This substantial gain confirms that the integrated model achieves a robust balance between Precision and Recall, effectively reducing both false alarms and missed detections. This significant leap demonstrates an effective synergistic mechanism. WIFA first acts as a filter to substantially suppress background noise and provide a clearer feature representation. Benefiting from this refined input, MKSS can more efficiently capture delicate morphological characteristics under reduced background distraction. Finally, CSFF robustly aligns and integrates these enhanced features across different scales. These three modules enhance the network from three different dimensions: frequency, space, and channel, forming the WMC-DFINE model.

As shown in Table 7, each proposed module yields positive gains for specific types of defect characteristics. Specifically, the WIFA module effectively improves detection metrics for categories susceptible to background interference such as NC and OP, while the MKSS module brings robust performance enhancements to scale-variant categories including SC and JF. Furthermore, the CSFF module demonstrates strong feature fusion capabilities in complex categories requiring contextual integration like DP. Through the synergistic effect of these three modules, the final WMC-DFINE model achieves stable performance improvements across all defect types.

Finally, we use WMC-DFINE as the teacher model to guide a student model with halved channel width through knowledge distillation. The resulting WMC-DFINE-distill has a parameter count of only 5.32 M and a computational overhead of 11.64 GFLOPs. Compared with the original DFINE, the distilled model achieves better performance while reducing the model size by nearly 50%, with mAP@0.5 stabilizing at 79.5%, offering an effective lightweight solution for real-time industrial inspection scenarios.

**Figure 11:**
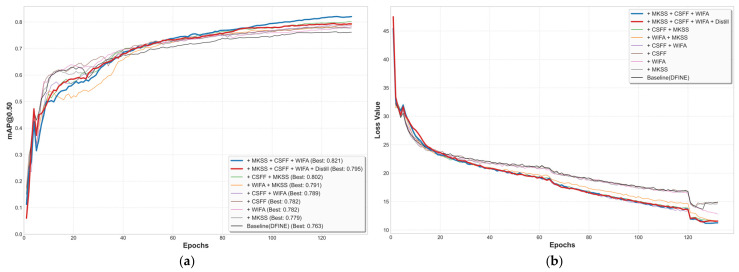
Training dynamics of the ablation experiments: (**a**) mAP@0.50 curves and (**b**) training loss curves.

Figure 11 depicts the learning curves of the models throughout the training process. Regarding the loss curves, the WMC-DFINE model represented by the bold blue line achieved the lowest loss value and exhibits the fastest convergence rate, which substantiates the stable training efficacy derived from the synergistic interaction of multiple modules. The loss for the distilled model WMC-DFINE-distill denoted by the bold red line remained slightly higher than that of the teacher model. This phenomenon is attributed to the increased optimization complexity arising from the dual constraints of ground-truth supervision and imitation learning. Furthermore, the abrupt plummet in loss observed at Epoch 120 validates the effectiveness of the fine-tuning strategy where strong data augmentation is disabled to refine the model weights. Regarding detection precision, the WMC-DFINE model attained the highest mAP and intuitively showcased the cumulative performance gains contributed by the integration of the WIFA, MKSS, and CSFF modules. Meanwhile, the distilled version WMC-DFINE-distill ultimately reached an mAP of 0.795, which is significantly superior to the baseline DFINE model, which oscillates at a lower accuracy level. This comparison effectively demonstrates that the student model has successfully inherited the sophisticated feature extraction fusion abilities from the teacher network through the knowledge distillation process.

**Figure 12:**
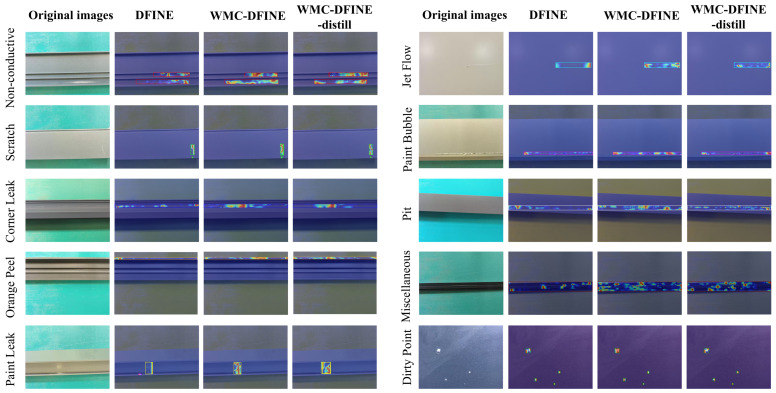
Attention heatmap visualization of the DFINE, WMC-DFINE, and WMC-DFINE-distill models across ten aluminum defect categories.

To thoroughly verify the feature representation ability of our proposed framework, we conducte attention visualization across all ten categories of aluminum surface defects in the dataset. Figure 12 shows a horizontal comparison of the heatmap distributions generated by the baseline DFINE model, the improved WMC-DFINE model, and the lightweight student WMC-DFINE-distill model.

The baseline DFINE architecture exhibits inherent limitations when processing defects characterized by complex surface textures. Specifically, during the detection of Orange Peel and Miscellaneous, the thermal response areas of the baseline model are notably constrained. In stark contrast, the WMC-DFINE model demonstrates a superior focal capability and robust background suppression. When detecting defects such as Non-conductive areas and Pits, WMC-DFINE effectively filters out background noise. Simultaneously, when encountering slender and minute flaws, such as Scratches and Jet Flows, the attention maps of our improved model comprehensively cover the defect morphologies. Notably, despite its substantially reduced parameter count, the heatmap distribution of WMC-DFINE-distill remains highly consistent with that of the teacher model. The visual evidence in Figure 12 substantiates the efficacy of the knowledge distillation strategy. The student model successfully inherits the detection expertise of the teacher for hard samples, thereby ensuring high localization precision of defect features while achieving an extremely lightweight architecture.

### 3.7. Comparative Experiments

To evaluate WMC-DFINE, we compared it against mainstream detectors on the identical dataset using unified hyperparameters including a 640 × 640 input size, the AdamW optimizer, and identical data augmentation. To ensure a fair comparison, training epochs were tailored to the convergence characteristics of each architecture. The quantitative results of these comprehensive evaluations are presented in Table 8.

To systematically investigate the balance between detection precision and efficiency, our analysis primarily focuses on the mAP@0.5 metric, F1-Score, parameter count, and FLOPs.

The proposed WMC-DFINE model achieves the optimal performance among all the evaluated models, attaining an mAP@0.5 of 82.1%, which represents a significant enhancement of 5.8 percentage points over the baseline DFINE architecture. This substantial improvement is primarily attributed to the effective suppression of high-frequency texture noise in the aluminum background by the WIFA module, the precise capture of defects across varying dimensions by the MKSS module, and the rich cross-scale contextual information provided by the CSFF module. Furthermore, the recall metric increases from 70.7% in the baseline to 76.2%, demonstrating a significant reduction in the false negative rate. Consequently, the F1-Score reaches 79.9%, confirming that WMC-DFINE achieves a robust balance between Precision and Recall compared to other mainstream detectors.

As shown in Table 6, although the non-end-to-end detectors ranging from YOLOv5s to YOLOv12s have progressively incorporated various advanced aggregation modules into their network architectures, the mAP@0.5 metric exhibits a marginal increase from 71.7% to 72.3%, yielding an improvement of only 0.6 percentage points. In stark contrast, end-to-end detectors demonstrate a substantially higher performance ceiling. Although early RT-DETR models perform on par with the YOLO series, the DFINE series optimized for the DETR architecture yields distinctly superior results. The baseline DFINE model achieves an mAP@0.5 of 76.3%, whereas our proposed WMC-DFINE further elevates this metric to 82.1%. Furthermore, even when compared to the recently proposed domain-specific DBA-RT-DETR [33] with an mAP@0.5 of 78.7% and an F1-Score of 78.8%, our WMC-DFINE maintains a distinct performance advantage, validating its superior structural adaptability to complex aluminum textures. Non-end-to-end models, which rely on local receptive fields and anchor boxes, encounter a distinct performance bottleneck when processing complex aluminum surfaces with severe texture interference and low contrast. Conversely, end-to-end models equipped with global modeling capabilities possess more robust feature extraction and detection proficiencies. The low recall values of Faster R-CNN and Gold-YOLO indicate that traditional matching strategies often miss irregular defects; even the best-performing YOLOv11s only achieves a recall of 73.6%. While YOLO models offer rapid inference, their pursuit of higher precision increases computational complexity without proportional accuracy gains. Early RT-DETR models are burdened by a massive parameter count and computational complexity; however, the DFINE architecture has successfully streamlined these aspects. Ultimately, WMC-DFINE-distill achieves a highly competitive mAP of 79.5% and a robust F1-Score of 79.1%, while requiring a computational complexity of merely 11.64 GFLOPs and a parameter count of only 5.32 M.

**Figure 13:**
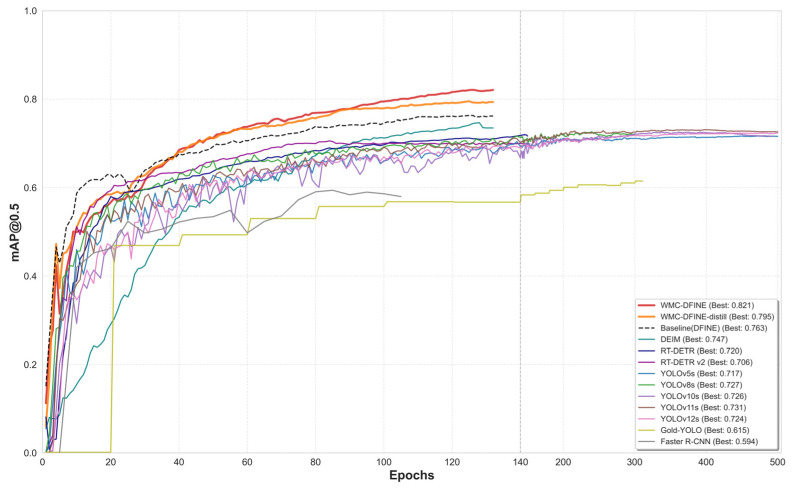
Training accuracy curves of the object detection models in the comparative experiments.

To gain deeper insights into the training progression of the models, we track their respective mAP@0.5 trajectories over the entire training phase. The resulting accuracy curves for all the comparative experiments are illustrated in Figure 13.

At the initiation of the training phase, the WMC-DFINE trajectory exhibits a remarkably steep ascent. This rapid progression indicates that the integration of the WIFA module enables the network to effectively suppress background interference from the outset, thereby preventing the model from engaging in redundant or ineffective feature extraction on complex aluminum textures. While most YOLO model curves gradually converge after Epoch 300, WMC-DFINE-distill maintains a slow yet continuous upward trajectory beyond Epoch 120, ultimately stabilizing around the 0.80 mark. This sustained growth illustrates that the distillation loss provides the student network with persistent soft-label guidance, successfully enabling it to escape local optima. A comparative analysis between the baseline DFINE represented by the black dashed line and the proposed WMC-DFINE denoted by the red solid line reveals that the improved architecture not only achieves a significantly higher performance ceiling but also exhibits superior robustness throughout the entire training regimen.

**Figure 14:**
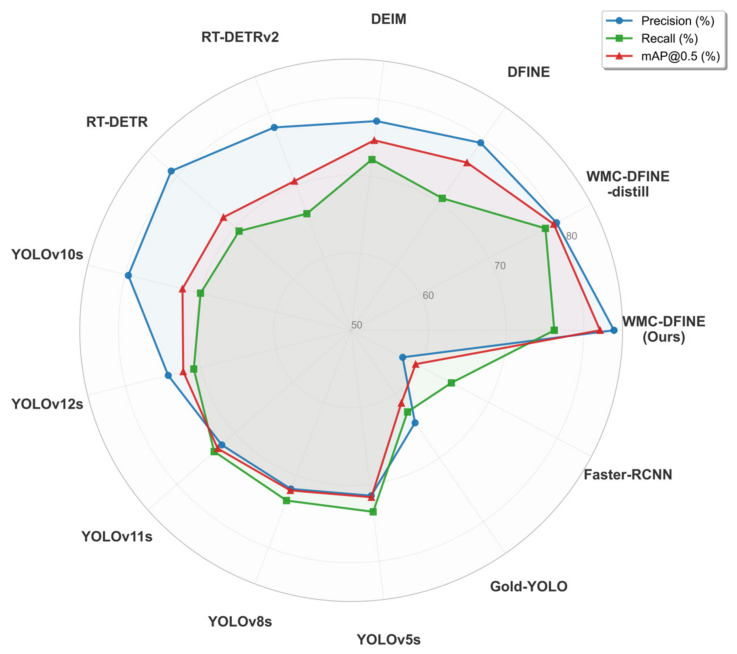
Multi-dimensional performance radar chart of the comparative experiments.

The multi-dimensional radar chart presented in Figure 14 provides a comprehensive visualization of the performance capabilities of the proposed approach. Among all evaluated detectors in the comparative experiments, WMC-DFINE architecture encompasses the largest closed polygonal area. This specific geometric morphology indicates that the model achieves an equilibrium across multiple evaluation metrics. Furthermore, the polygonal contour of the lightweight student WMC-DFINE-distill model closely tracks the geometric trajectory of its teacher counterpart, signifying that the lightweight student model not only inherits the accuracy metrics but also transfers the feature robustness of the teacher model.

**Figure 15:**
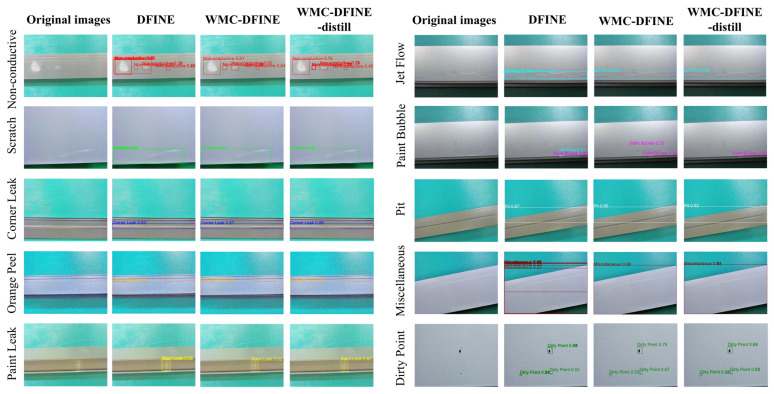
Inference visualization of the DFINE, WMC-DFINE, and WMC-DFINE-distill models on the test set.

To visually validate the model performance, we conduct an inference visualization on samples from the test set, as illustrated in Figure 15. The compared models include the baseline DFINE, WMC-DFINE, and WMC-DFINE-distill.

In the Dirty Point and Pit samples, the original DFINE model is prone to missed detections or inaccurate localization, whereas WMC-DFINE accurately bounds minute defects with higher confidence scores. In the Non-conductive and Orange Peel samples, the rough texture of the aluminum surface is highly susceptible to being misclassified as defects. As observed in the figure, the bounding boxes generated by WMC-DFINE and its distilled version are tighter, with extremely rare occurrences of background false positives. For elongated and extremely low-contrast defects, such as Scratches, the baseline model is prone to fragmented detections, while the WMC-DFINE model completely identifies the overall morphology of the scratch. Although the computational complexity of WMC-DFINE-distill is only 11.64 GFLOPs, its detection results approximate those of the teacher model. It maintains strong performance on complex defects, providing a new solution for the high-precision and real-time requirements of industrial sites.

## 4. Conclusions

To address the critical challenges of severe background texture interference, massive defect scale variations, and constrained computational resources in aluminum surface defect detection, in this study, we propose the WMC-DFINE end-to-end detection architecture and implement lightweight operations through model compression and channel distillation.

First, to mitigate the false positive issues caused by texture noise such as Orange Peel and Non-conductive anomalies, the WIFA module is integrated into the DFINE architecture. By decoupling features into the frequency domain and utilizing a gating mechanism to suppress high-frequency noise components, the model effectively separates the background from the defects. Second, to resolve the difficulties associated with minute Dirty Points and large-scale defects, the MKSS multi-kernel scale selection module and the CSFF cross-scale feature fusion module are proposed. The former preserves the feature details of varying defect sizes through dynamic receptive fields, whereas the latter guarantees feature continuity by establishing cross-level contextual dependencies. The synergistic interaction of these three modules elevates the mAP@0.5 of WMC-DFINE to 82.1%, surpassing current mainstream models, including YOLOv12s and RT-DETR. Furthermore, to meet the stringent requirements of high-speed industrial deployment, lightweight operations comprising model compression and channel distillation are executed. The experimental results demonstrate that the final WMC-DFINE-distill model not only compresses the parameter count to 5.32 M but also achieves a detection accuracy exceeding that of the original baseline model. This outcome provides a highly practical solution that simultaneously balances high precision and real-time efficiency for automated aluminum production lines based on machine vision sensing systems.

Despite its efficacy, the proposed method has certain limitations. First, the frequency-domain attention module lacks adaptability to complex non-stationary textures; future work will explore learnable wavelet transforms to address this. Second, to mitigate the occasional duplicate bounding boxes inherent to DETR architectures, we will investigate advanced deduplication strategies for extremely crowded scenarios. Finally, while validated on a challenging dataset, the model’s generalization across diverse material domains remains to be empirically evaluated. Our future work will include comprehensive cross-dataset validation to ensure cross-domain robustness under various manufacturing processes.

## Figures and Tables

**Table 1:** Detailed hardware and software configurations of the experimental environment.

Category	Component	Specification
Hardware	GPU	NVIDIA RTX 4090D (24 GB)
	CPU	Intel Xeon Platinum 8481C
	RAM	80 GB
	Storage	30 GB (System) 50 GB (Data)
Software	OS	Ubuntu 22.04 LTS
	Language	Python 3.12
	Framework	PyTorch 2.3.0
	Acceleration	CUDA 12.1

**Table 2:** Summary of hyperparameter settings employed during the training phase.

Title 1	Title 2	Title 3
General Settings	Input Resolution	640 × 640
	Total Batch Size	16
	Total Epochs	132
Optimizer (AdamW)	Initial Learning Rate	4 × 10^−4^
	Weight Decay	1 × 10^−4^
	Betas	(0.9, 0.999)

**Table 3:** Performance comparison of model scaling under different width multipliers.

Width Factor	Model	mAP@0.5 (%)	Params (M)	FLOPs (G)	FPS
1	DFINE	76.3	10.18	24.85	53.09
	WMC-DFINE	82.1	13.48	29.06	48.96
0.75	WMC-DFINE	79.2	9.34	18.97	55.34
0.50	WMC-DFINE	78.3	5.32	11.64	59.82
0.25	WMC-DFINE	75.5	2.82	7.06	64.15

**Table 4:** Experimental results of the knowledge distillation strategy.

Model	mAP@0.5 (%)	Params (M)	FLOPs (G)	FPS
DFINE	76.3	10.18	24.85	53.09
WMC-DFINE (Teacher)	82.1	13.48	29.06	48.96
WMC-DFINE-distill (Student)	78.3	5.32	11.64	59.82
WMC-DFINE-distill	79.5	5.32	11.64	59.75

**Table 5:** Validation of structural novelty and domain adaptation of the proposed modules.

Model	P (%)	R (%)	F1-Score (%)	mAP@0.5 (%)	Params (M)	FLOPs (G)
DFINE	79.4	70.7	74.8	76.3	10.18	24.85
DFINE+SE	79.6	72.3	75.8	77.0	10.25	24.91
DFINE+WIFA	78.9	79.5	79.2	78.2	13.01	27.17
DFINE+ InceptionNeXt	80.1	74.2	77.0	77.1	10.38	25.80
DFINE+MKSS	80.7	77.0	78.8	77.9	10.36	25.71
DFINE+CBAM	80.3	73.5	76.7	77.2	10.98	25.35
DFINE+CSFF	80.7	77.1	78.9	78.2	11.88	25.89

**Table 6:** Ablation study of the proposed modules on the aluminum surface defect dataset.

WIFA	MKSS	CSFF	Distill	P (%)	R (%)	F1-Score (%)	mAP@0.5 (%)	Params (M)	FLOPs (G)
				79.4	70.7	74.8	76.3 (±0.11)	10.18	24.85
√				78.9	79.5	79.2	78.2 (±0.19)	13.01	27.17
	√			80.7	77.0	78.8	77.9 (±0.18)	10.36	25.71
		√		80.7	77.1	78.9	78.2 (±0.12)	11.88	25.89
√	√			80.2	78.3	79.2	79.1 (±0.15)	13.57	30.24
√		√		82.1	79.2	80.6	78.9 (±0.17)	14.71	28.21
	√	√		80.1	78.2	79.1	80.2 (±0.13)	12.01	26.60
√	√	√		83.9	76.2	79.9	76.3 (±0.11)	13.48	29.06
√	√	√	√	79.9	78.3	79.1	78.2 (±0.19)	5.32	11.64

**Table 7:** Ablation study of the proposed modules across different defect categories.

Model	mAP@0.5 (%)									
	NC	SC	CL	OP	PL	JF	PB	PT	MI	DP
DFINE	83.5	38.7	99.5	86.4	96.2	50.5	33.8	99.4	96.5	78.5
+WIFA	88.6	37.1	99.5	92.2	95.8	50.2	34.6	99.4	96.8	87.8
+MKSS	82.9	46.1	99.5	85.1	96.8	52.8	35.8	99.5	97.1	83.4
+CSFF	85.2	39.8	99.5	88.0	97.0	51.6	36.8	99.5	98.1	86.5
+WIFA+MKSS	88.1	45.0	99.5	91.5	96.5	54.0	35.7	99.4	97.4	83.9
+WIFA+CSFF	90.4	37.5	99.5	92.8	97.2	52.0	36.4	99.5	98.0	85.7
+MKSS+CSFF	87.4	48.3	99.5	88.7	97.5	56.2	39.7	99.5	98.6	86.6
WMC-DFINE	92.9	48.6	99.5	95.8	98.1	58.4	42.8	99.4	97.9	87.6

(NC: Non-conductive; SC: Scratch; CL: Corner Leak; OP: Orange Peel; PL: Paint Leak; JF: Jet Flow; PB: Paint Bubble; PT: Pit; MI: Miscellaneous; DP: Dirty Point).

**Table 8:** Performance comparison of the proposed methods against mainstream object detectors on the aluminum surface defect dataset.

Model	P (%)	R (%)	F1-Score (%)	mAP@0.5 (%)	Params (M)	FLOPs (G)
	Non-end-to-end Real-time Object Detectors					
Faster-rcnn	57.5	64.6	60.8	59.4	136.8	145.7
YOLOv5s	71.5	73.6	72.5	71.7	9.12	23.8
YOLOv8s	71.9	73.5	72.7	72.1	11.12	28.5
Gold-YOLOs	64.5	62.8	63.6	61.4	21.5	46.5
YOLOv11s	72.3	73.6	72.9	73.0	9.41	21.3
YOLOv12s	74.3	70.9	72.6	72.3	9.23	22.7
	End-to-end Real-time Object Detectors					
YOLOv10s	79.6	70.0	74.5	72.4	7.22	21.4
RT-DETR	81.0	69.3	74.7	72.0	19.88	57.0
RT-DETRv2	78.0	66.1	71.6	70.6	19.89	59.89
DBA-RT-DETR	86.5	72.4	78.8	78.7	10.91	23.6
DEIM	77.2	72.2	74.6	74.7	10.18	24.85
DFINE	79.4	70.7	74.8	76.3	10.18	24.85
WMC-DFINE	83.9	76.2	79.9	82.1	13.48	29.06
WMC-DFINE-distill	79.9	78.3	79.1	79.5	5.32	11.64

## Data Availability

All original contributions of this study are incorporated in the present article, and further inquiries may be addressed to the corresponding authors.
